# Triiodothyronine (T3) increases the expression of the amphiregulin (AREG) oncogene by activating extranuclear pathways in MCF-7 breast cancer cells

**DOI:** 10.20945/2359-4292-2023-0094

**Published:** 2024-11-06

**Authors:** Maria Teresa De Sibio, Fernanda Cristina Fontes Moretto, Regiane Marques Castro Olimpio, Miriane de Oliveira, Lucas Solla Mathias, Vinícius Vigliazzi Peghinelli, Helena Paim Tilli, Bianca Mariani Gonçalves, Dariane Beatriz Marino Cardoso, Larissa Silva Dall Aqua, Igor de Carvalho Depra, Mariana Menezes Lourenço, Aline Carbonera Luvizon, Paula de Oliveira Montandon Hokama, Maria Tereza Nunes, Marna Eliana Sakalem, Célia Regina Nogueira

**Affiliations:** 1 Universidade Estadual Paulista Faculdade de Medicina de Botucatu Botucatu SP Brasil Universidade Estadual Paulista, Faculdade de Medicina de Botucatu, Botucatu, SP, Brasil; 2 Universidade de São Paulo Instituto de Ciências Biomédicas São Paulo SP Brasil Universidade de São Paulo, Instituto de Ciências Biomédicas, São Paulo, SP, Brasil; 3 Universidade Estadual de Londrina Departamento de Anatomia Londrina PR Brasil Departamento de Anatomia, Universidade Estadual de Londrina, Londrina, PR, Brasil

**Keywords:** Triiodothyronine, breast adenocarcinoma, MCF-7 cells, AREG, αvβ3 integrin

## Abstract

**Objective:**

Considering that the αvβ3 integrin plays an important role in tumor metastasis, this study investigated the involvement of these pathways in mediating the triiodothyronine (T3) effects on amphiregulin (*AREG*) expression.

**Materials and methods:**

We treated MCF-7 cells with T3 (10 nM) for 1 hour in the presence or absence of inhibitors for αvβ3 integrin (RGD peptide), MAPK (PD98059), PI3K (LY294002), and protein synthesis (cycloheximide [CHX]). A control group (C) received no T3 or inhibitors. Analyses of mRNA and protein expression were done using RT-qPCR and Western blot, respectively.

**Results:**

We observed that T3 increased *AREG* expression, an effect that was suppressed by all inhibitors. This finding indicates that the activation of the αvβ3 integrin signaling pathway, via PI3K, MAPK/ERK, is necessary for the T3-mediated effects on *AREG* expression and highlights the involvement of nongenomic mechanisms. In addition, CHX completely abolished T3-induced *AREG* mRNA expression, indicating that this effect requires prior protein synthesis.

**Conclusion:**

The identification that T3 acts through this signaling pathway holds considerable potential for clinical application, as it could lead to the development of specific drugs to block it.

## INTRODUCTION

The etiology of breast cancer is complex, involving both endogenous and exogenous factors that, when combined, can increase the risk of developing the disease (1). Metastasis of tumor cells is the primary cause of mortality among women with breast cancer (2). Experimental studies have found that thyroid hormones (THs), especially triiodothyronine (T3), can influence both the differentiation of healthy mammary cells and the proliferation of breast cancer cells (3-7). Although epidemiological studies offer contradictory explanations about the influence of T3 on breast cancer, reviews from our group have demonstrated its ability to increase the proliferation of cancer cells (3,6,8). As an example, DNA microarray studies performed in our laboratory using MCF-7 cells have shown that T3 treatment for 24 hours significantly increased the expression of eight genes, of which amphiregulin (*AREG*) was the most expressed (4). This is in accordance with studies from other groups that showed that T3 treatment increases AREG protein expression (2). Several clinical studies reported the importance of AREG in the progression of breast tumors, as well as its association with tumor aggressiveness and poor prognosis (8).

It is known that T3 can act independently of its classical nuclear pathway, which involves interaction with the TH receptor (THR)-TH responsive element (TRE) in TH-inducible genes (9). Such action is called extranuclear or nongenomic action. In this case, T3 may initiate its action on cell membrane or cytoplasm binding sites, activating a protein phosphorylation cascade, which triggers rapid effects on cells that can lead to gene expression modulation. The extranuclear pathways include the alpha-v beta-3 (αVβ3) integrin, mitogen-activated protein kinase (MAPK), and phosphatidylinositol 3-kinase (PI3K) pathways (10-14). Thus, T3 binding to the αvβ3 integrin at the plasma membrane activates the cytoplasmic MAPK and PI3K pathways (9,10,15). The protein extracellular signal-regulated kinase (ERK)1/2 is one of the kinases that participate in the MAPK pathway and, once activated inside the cytoplasm, phosphorylates target proteins that can modulate gene transcription. In a similar way, the activation of PI3K in the cytosol results in a subsequent stimulation of protein kinase B (AKT/PKB), which also affects gene expression (16).

Complex cellular events initiated at cell-surface receptors also include tumoral cell proliferation (17), in which αvβ3 integrin seems to play an important role. The present study aimed to examine whether the modulatory effect of T3 on AREG expression involves the activation of extranuclear signaling pathways, by exploring the role of the αvβ3 integrin signaling pathways in this effect using MCF-7 human breast cancer cell line.

## MATERIALS AND METHODS

### Reagents and antibodies

Fetal bovine serum (FBS), RPMI, and antibiotic solution (100×) were purchased from Life Technologies Corporation (Grand Island, NY, USA). Cycloheximide (CHX), LY294002 (LY), PD98059 (PD), and GRGDSP peptide were purchased from Proteimax (Cotia, SP, Brazil). Dimethylsulfoxide (DMSO), T3, sodium hydroxide (NaOH), and charcoal-stripped FBS were purchased from Sigma-Aldrich (St. Louis, MO, USA). Mouse monoclonal anti-amphiregulin [MM0089-8K16] (ab89119) and rabbit monoclonal anti-GAPDH (ab181602) antibodies were acquired from Abcam (Cambridge, UK). Anti-mouse antibody and anti-rabbit secondary antibodies were purchased from Santa Cruz Biotechnology (Santa Cruz, CA, USA). Enhanced chemiluminescence (ECL) reagents were obtained from Amersham Biosciences (Piscataway, NJ, USA).

### Cell culture

The MCF-7 cell line (breast cancer immortalized cells) was initially derived from a primary culture of breast cancer cells originating from the pleural effusion of a female patient with metastasis (18). These cells express ER1, ER2, and THR α and β (19,20). This cell line was purchased from American Type Culture Collection (ATCC; Manassas, VA, USA). All experiments were performed in triplicate for all treatments and times, and the MCF-7 cells were used between the second and third passages.

The MCF-7 cells were cultivated in RPMI 1640 medium supplemented with 1.2 g/L NaHCO3, 10 mM HEPES, pH 7.4, and 10% FBS, and maintained at 37 °C in 5% CO_2_. The medium was changed every 2 days. Before starting the drug and hormone treatments, the medium was replaced with a phenol red-free medium to remove all hormones present. The cells were then incubated with 10% charcoal-stripped FBS for 24 hours. After incubation, the cells were treated with 10 nM T3 or vehicle (0.1% NaOH; control group [C]) for 10 minutes, 30 minutes, 1 hour, and 4 hours, in order to define the time points to be evaluated in the subsequent experiments, with inhibitor treatments. Based on these results, the 1-hour time point was selected for all subsequent experiments. The T3 dose of 10 nM has been extensively used by our research group, yielding successful results (12,14,21-24). The cells were incubated with CHX (50 μM), LY294002 (50 μM), PD98059 (50 µM), or Arg-Gly-Asp (RGD) peptide (100 µg/mL) 1 hour prior to T3 treatment. All treatments started simultaneously, and the cells were collected at each time point.

### Gene expression

Total RNA was extracted from the MCF-7 cells using TRIzol (Invitrogen, São Paulo, Brazil), according to the manufacturer's instructions. The High-Capacity cDNA Reverse Transcription Kit for RT-PCR (Invitrogen, São Paulo, Brazil) was used to synthesize single-stranded DNA (ssDNA) from total RNA. Levels of *AREG* expression (Hs00950669_m1; Thermo Fisher Scientific Inc., Waltham, MA, USA) were determined by quantitative reverse transcription polymerase chain reaction (RT-qPCR) using the StepOne Plus system with the TaqMan qPCR commercial kit (Applied Biosystems, Waltham, MA, USA), according to the manufacturer's instructions. The amplification conditions were as follows: activation of the enzyme at 50 °C for 2 minutes, denaturation at 95 °C for 10 minutes, and cDNA amplification during the 40 cycles comprising denaturation at 95 °C for 15 seconds and extension at 60 °C for 1 minute. All experiments were performed in duplicate. Gene expression was quantified relative to the data obtained from the control group, after normalization with an internal control (GAPDH, Hs02758991_g1; Thermo Fischer Scientific Inc.), using the 2-ΔΔCt method, as described previously (25).

### Western blot analysis

After treatment with T3 and inhibitors, cells were washed twice with cold PBS and lysed with 100 μL of lysis buffer containing 500 mM Tris (pH 8), 150 mM NaCl, 1% Triton X-100, 0.1% sodium dodecyl sulfate (SDS), and 0.5% sodium deoxycholate, as previously described. The homogenate was centrifuged at 4 °C for 20 minutes at 12,000 rpm. The supernatant was collected, and total protein content was determined by the Bradford method, using Bovine Serum Albumin (BSA) as the standard. Samples containing 50 μg of protein were subjected to 15% SDS-polyacrylamide gel electrophoresis (SDS-PAGE), and proteins were electrotransferred to a nitrocellulose membrane using a semidry transfer system (Bio-Rad Laboratories, Inc., Hercules, CA, USA). The blotted membrane was then blocked with 5% BSA in tris-buffered saline (TBS) plus 0.1% Tween 20, for 1 hour at room temperature and incubated with specific antibodies overnight at 4°C. The primary antibodies used were rabbit polyclonal anti-amphiregulin 1:1000 [MM0089-8K16] (ab89119), rabbit monoclonal anti-GAPDH 1:200 (ab181602), anti-mouse antibody, and anti-rabbit secondary antibodies. After incubation with secondary antibody anti-rabbit (1:2000) in 5% milk in TBS-T for 1 hour at room temperature, blots were developed using the SuperSignal West Pico Chemiluminescent Substrate kit (Pierce Biotechnology, Rockford, IL, USA) and exposed to Hyperfilm ECL (Amersham Biosciences). Quantitative analysis of immunoblot images was performed using Gel Logic (6000 PRO) with Carestream MI software (Carestream, Rochester, NY, USA). The results are shown as the ratio of the squared pixel intensities between amphiregulin and GAPDH, expressed as fold activation relative to cells exposed to the assay media.

### Statistical analysis

Gene and protein expressions were assessed using analysis of variance (ANOVA) followed by Tukey's test, after the data passed the normality test. Data are expressed as mean ± standard deviation. The significance level was set at 5%.

## RESULTS

### T3 affects *AREG* expression in MCF-7 cell lines

**Figure 1:**
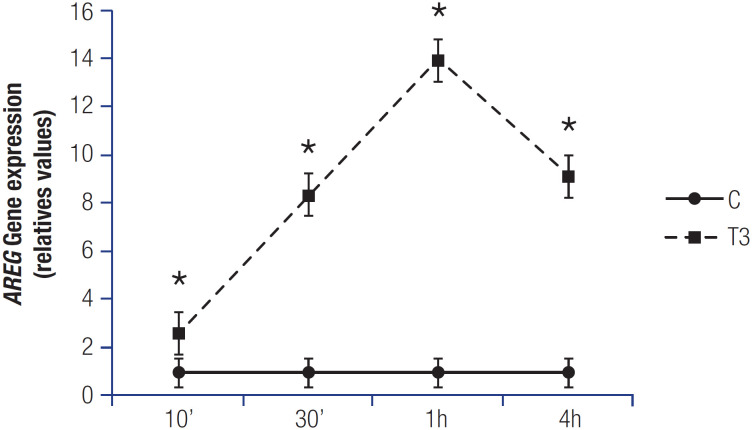
Time-dependent effect of T3 (10 min, 30 min, 1 h, and 4 h) on *AREG* mRNA expression in MCF-7 cells. T3 = 10 nM, C = untreated cells (vehicle). Data are expressed as mean ± standard deviation and were analyzed using Tukey's test. *P < 0.05 for the C versus T3 comparison. N = 3 for each treatment.

Treatment with T3 increased the expression levels of *AREG* mRNA in MCF-7 cells (Figure 1) after 10 minutes, 30 minutes, 1 hour, and 4 hours of incubation compared with no treatment (control group [C]).

### The increase in T3-induced *AREG* expression is prevented by the RGD peptide (an αvβ3 integrin pathway inhibitor)

**Figure 2:**
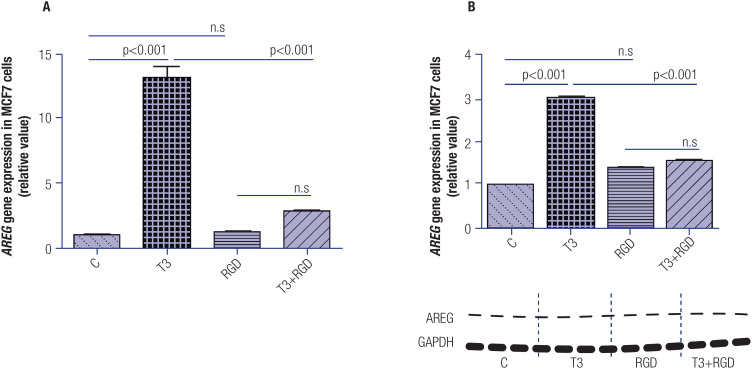
Effect of T3 (1 h) and RGD peptide (RGD) treatment on the modulation of *AREG* expression levels in MCF-7 cells. Panels **A** and **B** illustrate the effect of T3 on *AREG* mRNA and AREG protein expressions, respectively, after blocking of αvβ3 integrin activity. Below the graph in panel **B** is a typical autoradiogram representative of AREG and GAPDH. T3 = 10 nM, T3 + RGD = T3 10 nM plus RGD 5.0 μg/mL. Data are expressed as mean ± standard deviation and were analyzed using analysis of variance (ANOVA) supplemented by Tukey's test. N = 3 for each treatment. N.s = nonsignificant.

To determine the role of the activation of the αvβ3 integrin pathway on the regulation of T3-induced *AREG* expression, we incubated the MCF-7 cells with RGD for 1 hour, followed by incubation with T3 for another 1 hour. Figures 2A and 2B show that the inhibition of the αvβ3 integrin pathway abrogated the T3-induced *AREG* gene and AREG protein expressions, respectively, in the MCF-7 cell lines.

### The T3-induced increase in *AREG* expression is dependent on PI3K activity

**Figure 3:**
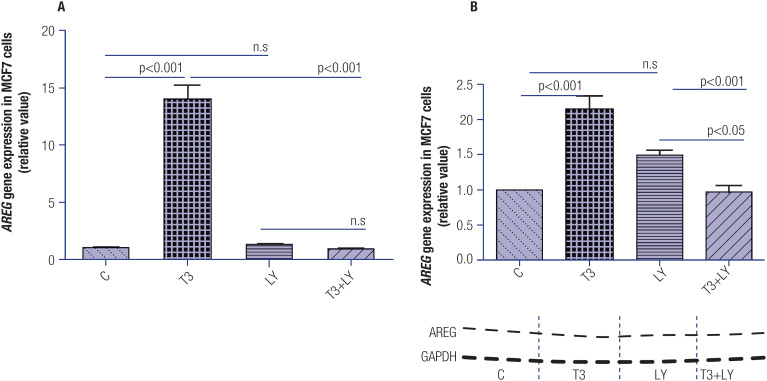
Effect of T3 treatment (1 h) on the modulation of *AREG* expression in MCF-7 cells in the presence or absence of LY294002 (LY). Panels **A** and **B** illustrate the effect of T3 on *AREG* mRNA and AREG protein expressions, respectively, after blocking of PI3K activity. Below the graph in panel **B** is a typical autoradiogram representative of AREG and GAPDH. T3 = 10 nM, T3+ LY= T3 10 nM plus LY294002 50 μM. Data are expressed as mean ± standard deviation and were analyzed using analysis of variance (ANOVA) supplemented by Tukey's test. N = 3 for each treatment. N.s = nonsignificant.

To determine the role of the activation of the PI3K pathway in the regulation of T3-induced *AREG* expression, we incubated MCF-7 cells for 1 hour with LY294002, followed by incubation with T3 for another 1 hour. Figures 3A and 3B show that the inhibition of the PI3K pathway prevented the increase in *AREG* mRNA and AREG protein expressions induced by T3, respectively.

### The increase in T3-induced *AREG* expression is modulated by the MAPK/ERK pathway

**Figure 4:**
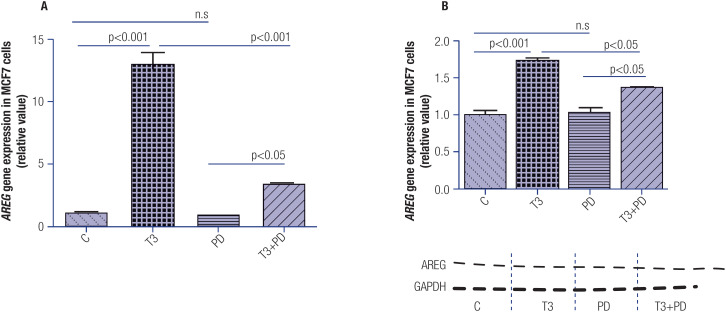
Effect of T3 (1 h) and PD98059 (PD) treatment on the modulation of *AREG* expression levels in MCF-7 cells. Panels **A** and **B** illustrate the effect of T3 on *AREG* mRNA and AREG protein expressions, respectively, after blocking of MAP/ ERK activity. Below the graph in panel **B** is a typical autoradiogram representative of AREG and GAPDH. T3 = 10 nM, T3 + PD = T3 10 nM plus PD 5.0 μg/mL. Data are expressed as mean ± standard deviation and were analyzed using analysis of variance (ANOVA) supplemented by Tukey's test. N = 3 for each treatment. N.s = nonsignificant.

To determine the role of the activation of the MAPK/ERK pathway in regulating the T3-induced *AREG* expression, we incubated MCF-7 cells for 1 hour with PD98059, an inhibitor of this pathway, followed by incubation with T3 for 1 hour. Figures 4A and 4B show that the inhibition of the MAPK/ERK pathway led to a decrease in the T3-induced *AREG* mRNA and AREG protein expressions, respectively, in the MCF-7 cell lines.

### Effect of the cycloheximide-mediated inhibition of protein synthesis on the T3-mediated expression of *AREG* in MCF-7 cell lines

**Figure 5:**
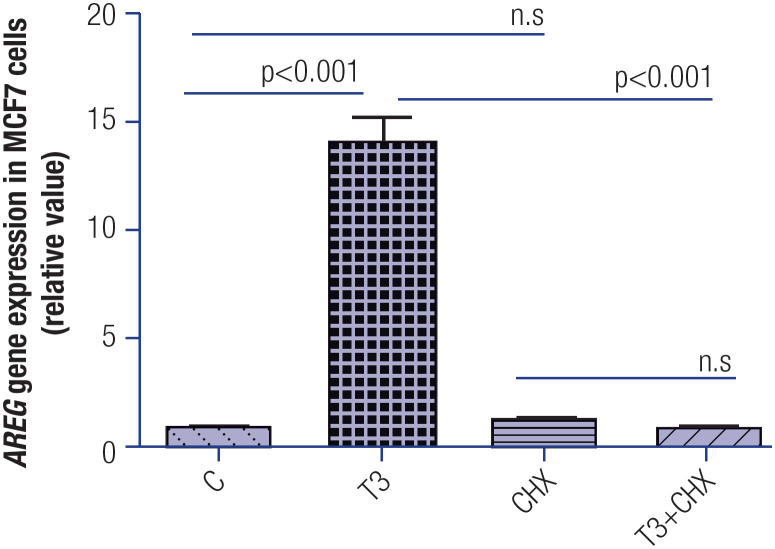
Effect of T3 treatment (1 h) on the modulation of *AREG* mRNA levels in MCF-7 cells in the presence or absence of cyclohexamide (CHX). T3 = 10 nM, T3+ CHX = T3 10 nM plus CHX 50 μM. Data are expressed as mean ± standard deviation and were analyzed using analysis of variance (ANOVA) supplemented by Tukey's test. N = 3 for each treatment. N.s = nonsignificant.

To determine whether protein synthesis is necessary for the increased *AREG* mRNA expression triggered by T3, MCF-7 cells were incubated in the presence or absence of CHX for 1 hour and subsequently treated with T3 for 1 hour. Figure 5 shows that the inhibition of protein synthesis by CHX abrogated the T3-induced *AREG* expression in MCF-7 cells.

## DISCUSSION

A positive association between breast cancer incidence and serum T3 levels has been previously reported, even after menopause (26,27). Previous investigations have demonstrated that patients with breast cancer have higher free T3 (and T4) levels and lower TSH levels compared with age-matched individuals (28). Notably, T3 regulates genes associated with cancer cell invasion (3,6,7), including *AREG*, which is involved in the proliferation and branching of various tissues. These effects are mediated by the induction of genes related to cell invasion and migration. Additionally, *AREG* has been implicated in breast cancer progression and aggressiveness and has been identified as a key target gene for T3 in MCF-7 breast cancer cells (4,5,8,19). In the present study, we observed an increased *AREG* mRNA expression in MCF-7 cells after T3 treatment, at the dose of 10 nM after 10 minutes, 30 minutes, 1 hour, and 4 hours (Figure 1). This characterizes a short-term effect of T3, according to Figueiredo and cols. (4), who demonstrated that *AREG* is modulated by T3 in the same cell line, but after a longer period of time (24 hours). In our data, after every tested time, the same behavior was observed in relation to *AREG* expression in response to T3; as a consequence, the treatment period (exposure time to T3) of 1 hour was used as a standard for the subsequent experiments.

Considering that T3 can activate extranuclear pathways, such as the ones related to membrane αvβ3 integrin as well as the cytoplasmic MAPK/ERK and PI3K/AKT, we investigated whether the increased *AREG* expression induced by T3 depends on the activation of these pathways. For this purpose, an αvβ3 antagonist – the Arg-Gly-Asp (RGD) peptide – was added to inhibit the αvβ3 integrin pathway. The results showed that exposure of MCF-7 cells to RGD led to inhibition of the T3-induced *AREG* expression, reinforcing the importance of T3 binding to αvβ3 integrin to trigger its carcinogenic effect (Figures 2 A and 2B). In this context, αvβ3 integrin has been considered a key organizer that assembles fibronectin, fibrinogen, proteolyzed collagen, and tenascin into a complex to induce cell migration. If αvβ3 integrin is inactivated, angiogenesis and cell migration are inhibited (29); αvβ3 integrin also binds to different ligands to regulate cellular proliferation and metastasis (30). The RGD peptides engage with integrins and can be successfully employed in cancer treatments since they help target cancer cells and tumorous vasculature, aiding drug delivery and reducing adverse effects on healthy tissues (31). Integrins are a family of heterodimeric glycoproteins that act as cell-surface receptors and are composed of a larger α subunit and a smaller β subunit. The multiple α and β subunits present in mammals (18 and 8, respectively) can combine to form 24 distinct αβ integrin pairs. Integrins present both specific and overlapping ligand-binding activity, and through formed complexes, they transduce complex signals into the cytoplasm and regulate multiple biological events (32). Notably, THs can bind to integrins’ subunits, particularly the β (or S1) subunits, which bind exclusively to T3 and activate the PI3K pathway through Src kinase. The α subunits, also known as S2, bind to T3 or T4 but present higher affinity to T4, and activate the MAPK/ERK1/2 pathway (33,34).

The activation of PI3K in the cytoplasm catalyzes the conversion of phosphatidylinositol 4,5-bisphosphate (PIP2, phosphatidylinositol [4,5]-bisphosphate) into phosphatidylinositol 3,4,5-trisphosphate (PIP3, phosphatidylinositol [3,4,5]-trisphosphate), which transduces a signal by interacting with the serine/threonine kinase AKT (PKB) (35). The PI3K/AKT pathway is an important signaling pathway for many cellular functions, such as growth control, metabolism, and translation initiation. This pathway has many components, and their inhibition can lead to tumor suppression, making them promising candidates for anti-cancer drugs (36) and targeted therapy (37). The PI3K inhibitor LY294002 is a medication that inhibits one or more of the PI3K enzymes, regulating mitochondrial function and potentially alleviating certain types of cancer (38,39).

The interruption of upstream AKT signaling through the PI3K inhibitor LY294002 abolished the increase in *AREG* gene and AREG protein expressions in T3-treated MCF-7 cells, strongly suggesting the involvement of the PI3K signaling pathway in the T3-induced upregulation of *AREG* (Figures 3A and 3B). It is noteworthy that the association of inhibitors of the PI3K pathway with endocrine therapy in advanced hormone receptor-positive breast tumors has been shown to improve cytotoxic responses and prevent resistance (40), reinforcing the results obtained by our group. Considering the critical role of the MAPK/ERK pathway in cancer development (41), and that T3 may activate this pathway by an extranuclear mechanism (9)we determined the role of MAPK/ERK pathway activation in regulating *AREG* gene expression induced by T3, by incubating MCF-7 cells with PD98059. This demonstrates that the inhibition of the MAPK/ERK pathway reduces *AREG* gene expression induced by T3 in this breast cancer cell line (Figures 4A and 4B).

The MAPK/ERK pathway can be activated by T3 in the cell membrane or directly in the cytoplasm, initiating a phosphorylation cascade. After the transmembrane receptor activation by T3, there is a phosphorylation of RAS protein induced by the recruitment of adaptative molecule growth factor receptor-bound protein 2 (GRB2) and a trade factor of guanine nucleotide (SOS). This event is followed by a sequential stimulation of diverse cytoplasmic kinase proteins, such as Raf (a specific kinase for AKT), which directly activates MEK and MAPKs, and among them, ERK1/2. On the other hand, MAPKs migrate to the cellular nucleus and phosphorylate a set of molecules that are responsible for cell transcription, which triggers several cellular functions (42). Notably, PD98059 is a highly selective *in vitro* inhibitor of MEK1 activation and the MAPK cascade (43); it binds to inactive forms of MEK1, preventing their activation (44). The RAS-ERK and PI3K-phosphatidylinositol 3 kinase-mammalian target of rapamycin (mTOR) signaling pathways are the cell's chief mechanisms for controlling cell survival, differentiation, proliferation, metabolism, and motility in response to extracellular cues; the activation of RAS increases some pathways, controlled by PI3K-mTOR, that induce growth, and there is a strong link between metabolic rewiring in cancer and such pathways (45).

To determine whether protein synthesis was required for the T3 modulation of the *AREG* expression, we used CHX, an inhibitor of protein synthesis. This inhibitor prevented the increase in *AREG* mRNA levels induced by T3 in MCF-7 cells (Figure 5). Therefore, the effect of T3 depends on protein synthesis. To our knowledge, this is the first study to show a rapid and indirect action of T3 increasing mRNA levels of *AREG* in breast cancer cells. Even though AREG has not demonstrated the high sensitivity or specificity required for its use as a biomarker for early prognosis of breast cancer in a population (46), high AREG levels are able to confer resistance to trastuzumab treatment, probably by promoting sustained phosphorylation of the PI3K/AKT and MAPK/ERK pathways, even after the use of this drug (47).

In conclusion, the results of this study demonstrate that T3 increases *AREG* expression by activating αvβ3 integrin, MAP/ERK, and PI3K signaling pathways and that the inhibition of the protein synthesis abolishes the effects of T3 on *AREG*. These events characterize an extranuclear action of T3, dependent on protein synthesis. The findings of this study are of chief importance, considering that *AREG* is implicated in the processes of disease progression and poor prognosis. These data present a high potential for application, as the identification of new signaling pathways and other mechanisms by which action on THs occurs could lead to the development of specific drugs that block these pathways, promoting desirable effects while blocking undesirable ones.
